# Supplementary material for: Targeting NAD+ regeneration enhances antibiotic susceptibility of Streptococcus pneumoniae during invasive disease
Source: PLoS Biol. 2023 Mar 16;21(3):e3002020. doi: 10.1371/journal.pbio.3002020 (PMC10019625; doi:10.1371/journal.pbio.3002020)

A)

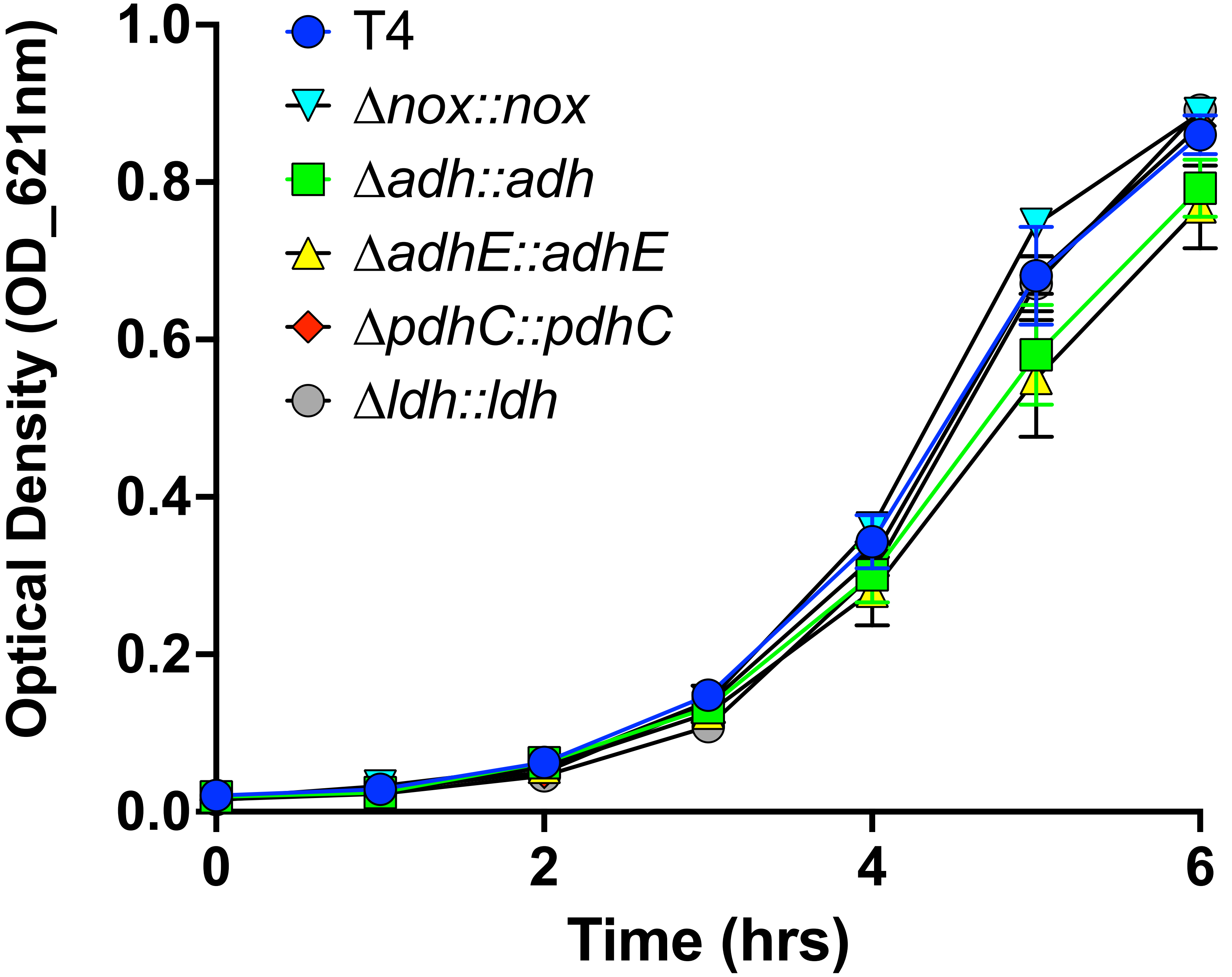

Spn TIGR4	T4	Δ*nox* ::*nox*	Δ*adh* ::*adh*	Δ*adhE* ::*adhE*	Δ*pdhC* ::*pdhC*	Δ*ldh* ::*ldh*
Doubling Time(min)	56.5 ± 1.6	54.0 ± 1.2	57.5 ± 3.0	58.3 ± 4.6	52.6 ± 3.0	53.2 ± 0.8

B)

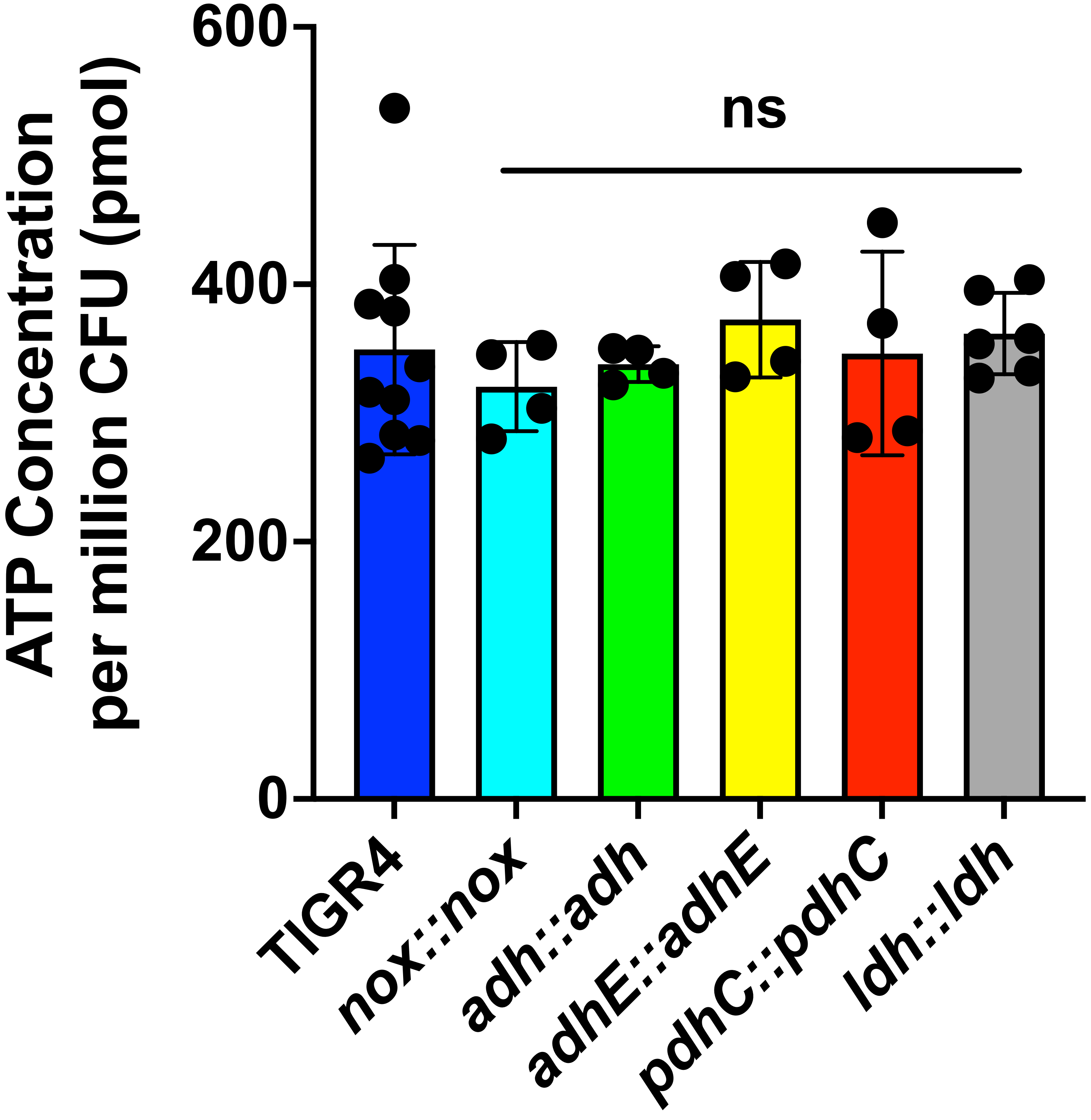

Supplement: S1 Fig — (A) Growth rate of the Spn TIGR4 and revertant strains in THY media. The growth rate was examined every hour for 6 hours (n = 4). (B) Intracellular ATP concentration of Spn TIGR4 and revertant strains (n ≥ 4). The data underlying these figures can be found in S1 Data. Statistical analyses were done using the Mann–Whitney t test and one-way ANOVA. (PDF) [file pbio.3002020.s001.pdf]
