# Supplementary figures and images for: Targeting NAD+ regeneration enhances antibiotic susceptibility of Streptococcus pneumoniae during invasive disease
Source: PLoS Biol. 2023 Mar 16;21(3):e3002020. doi: 10.1371/journal.pbio.3002020 (PMC10019625; doi:10.1371/journal.pbio.3002020)

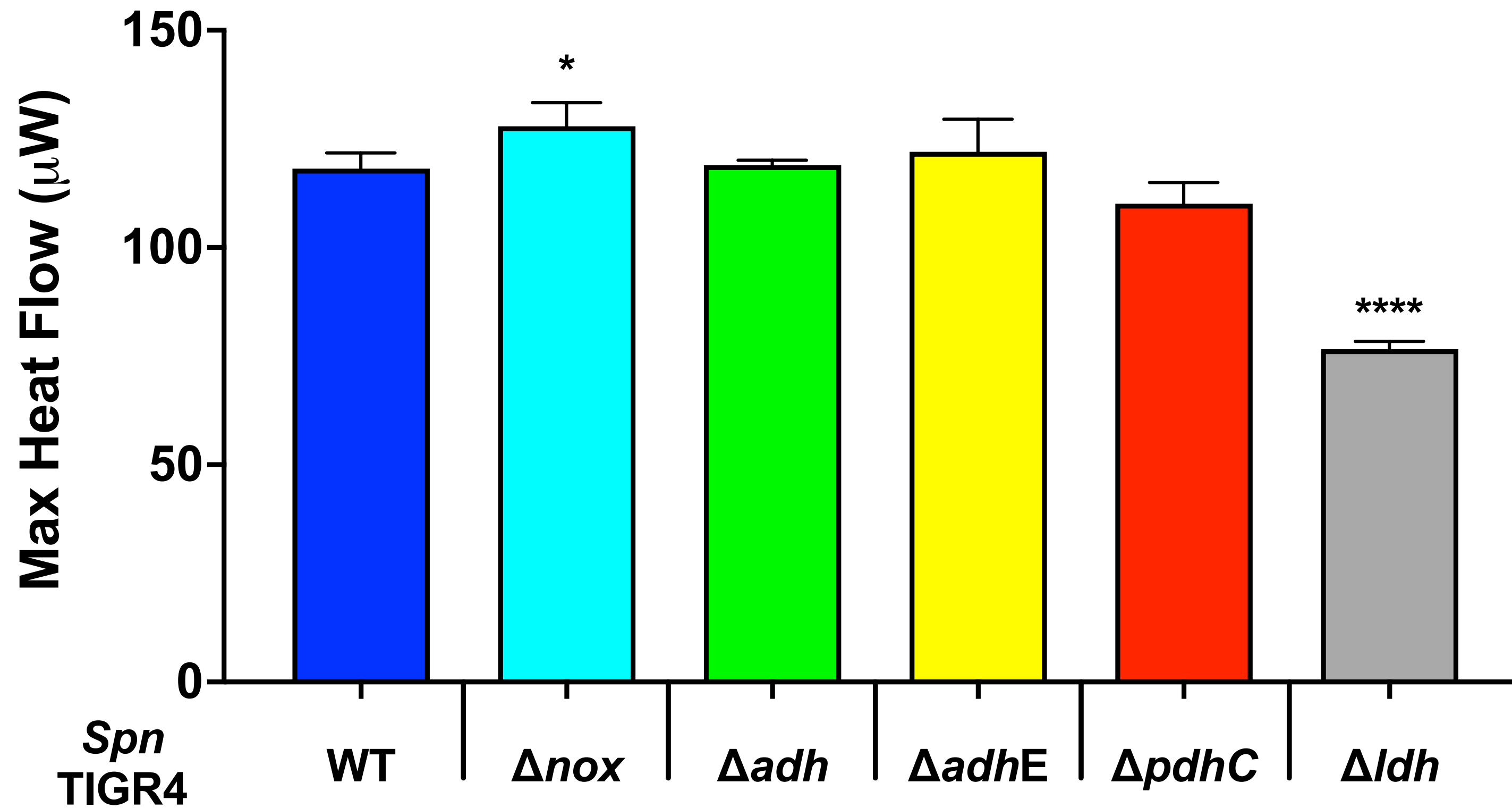

Supplement: S2 Fig — Maximal heat flows of Spn wild type and its isogenic mutants involved in NAD+ regeneration during 24 hours of the cultivation. The measurement was made using the calorimeter. The data underlying this figure can be found in S1 Data. Statistical analyses were done using one-way ANOVA with the Dunnett post hoc test (n ≥ 3). (PDF) [file pbio.3002020.s002.pdf]

## Lactate Dehydrogenase

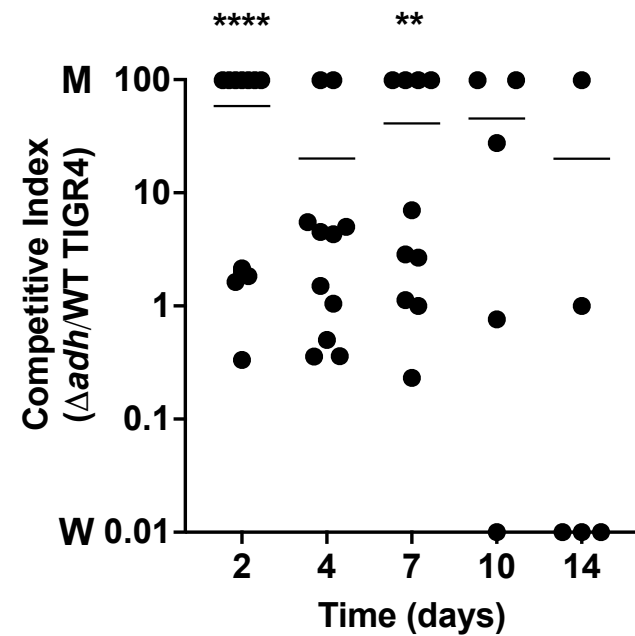

## Alcohol Dehydrogenase

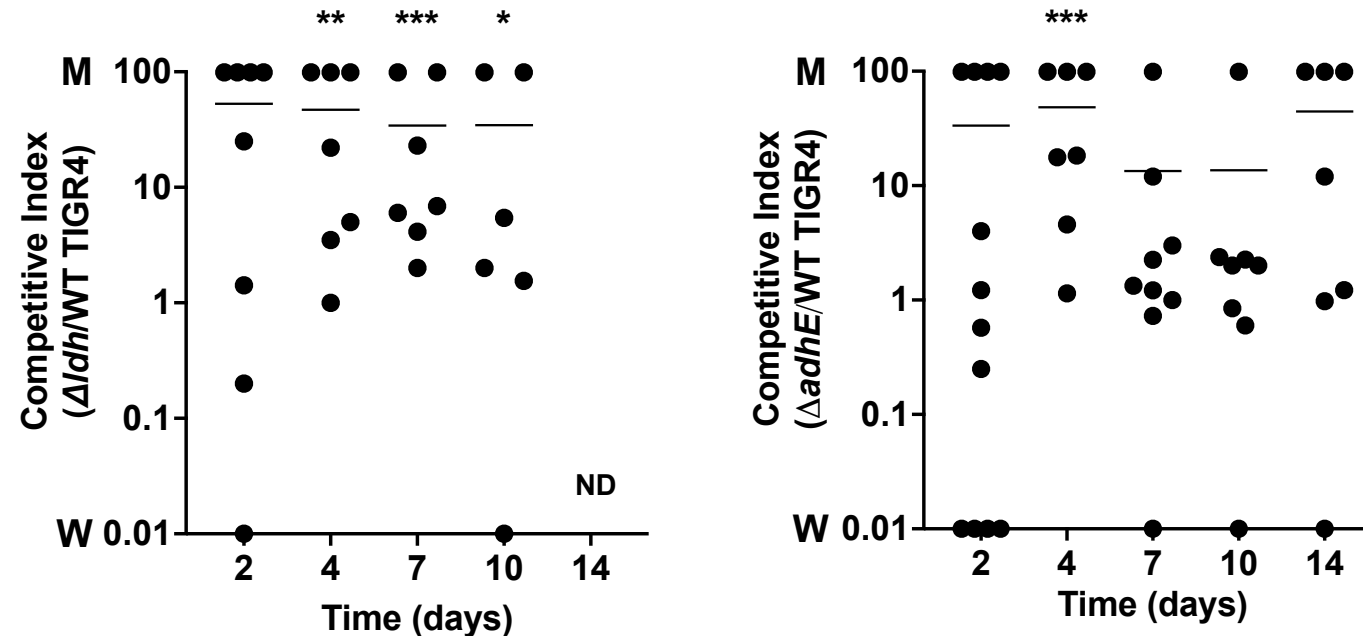

## Pyruvate Dehydrogenase

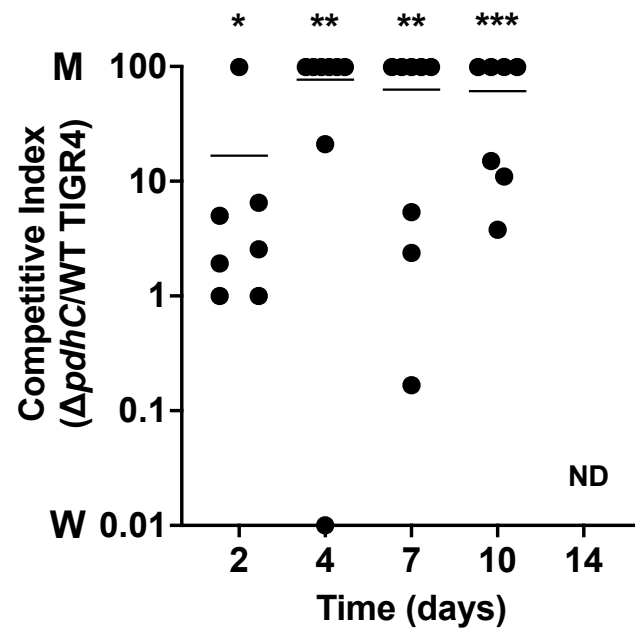

## NADH Oxidase

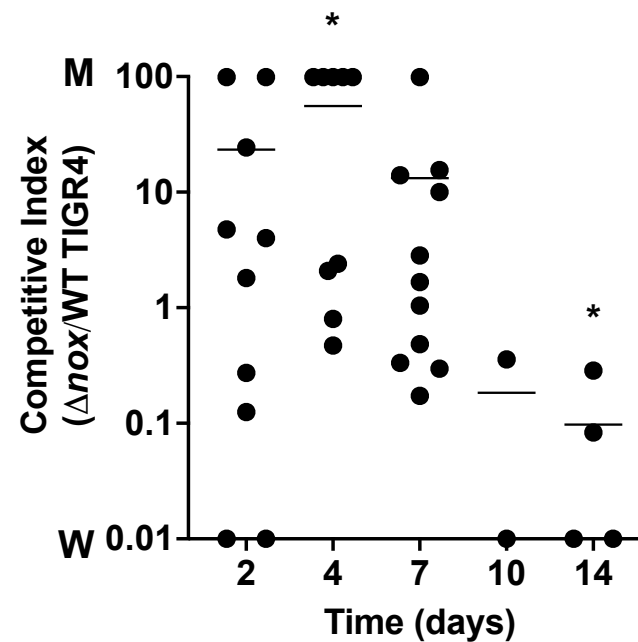

Supplement: S3 Fig — For nasopharyngeal colonization, mice were colonized intranasally 1 × 10^6^ CFU of a 1:1 ratio mixture of Spn TIGR4 and mutant in 10 μl volume. The bacterial number was evaluated in the colonization model by a nasal wash at 2, 4, 7, 10, and 14 days after colonization. The number of mutants were enumerated using kanamycin as a selection marker. Statistical analyses were done using the Mann–Whitney t test. The data underlying this figure can be found in S1 Data. Asterisks indicate statistical significance: *, P ≤ 0.05, **, P ≤ 0.01; ***, P ≤ 0.001; ****, P ≤ 0.0001. (PDF) [file pbio.3002020.s003.pdf]

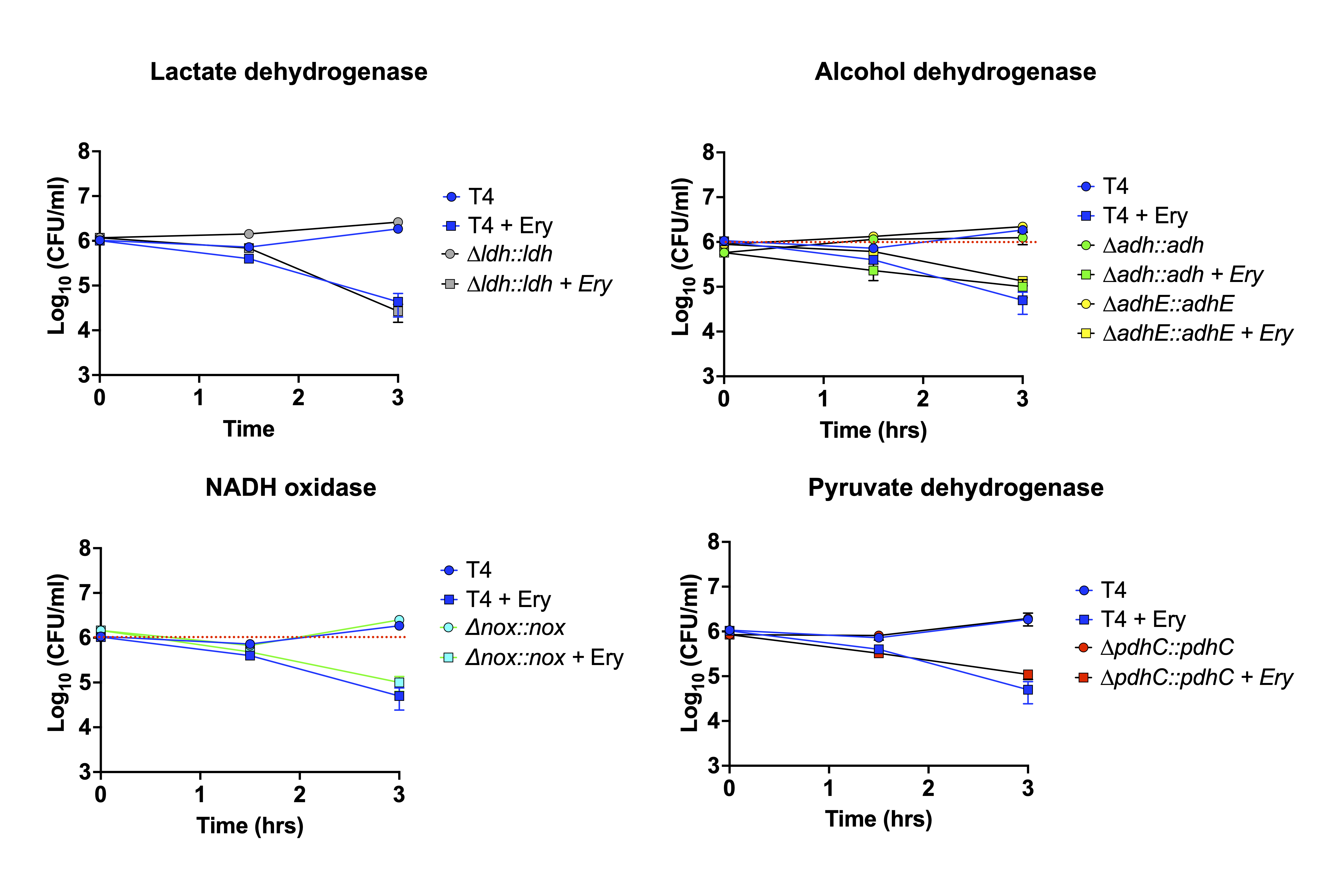

Supplement: S4 Fig — The viability of Spn TIGR4 and revertant strains was measured after 90 and 180 minutes of exposure with and without erythromycin (50 μg/ml). The data underlying this figure can be found in S1 Data. (n ≥ 3) Statistical analyses were done using the Mann–Whitney t test. (TIFF) [file pbio.3002020.s004.tiff]

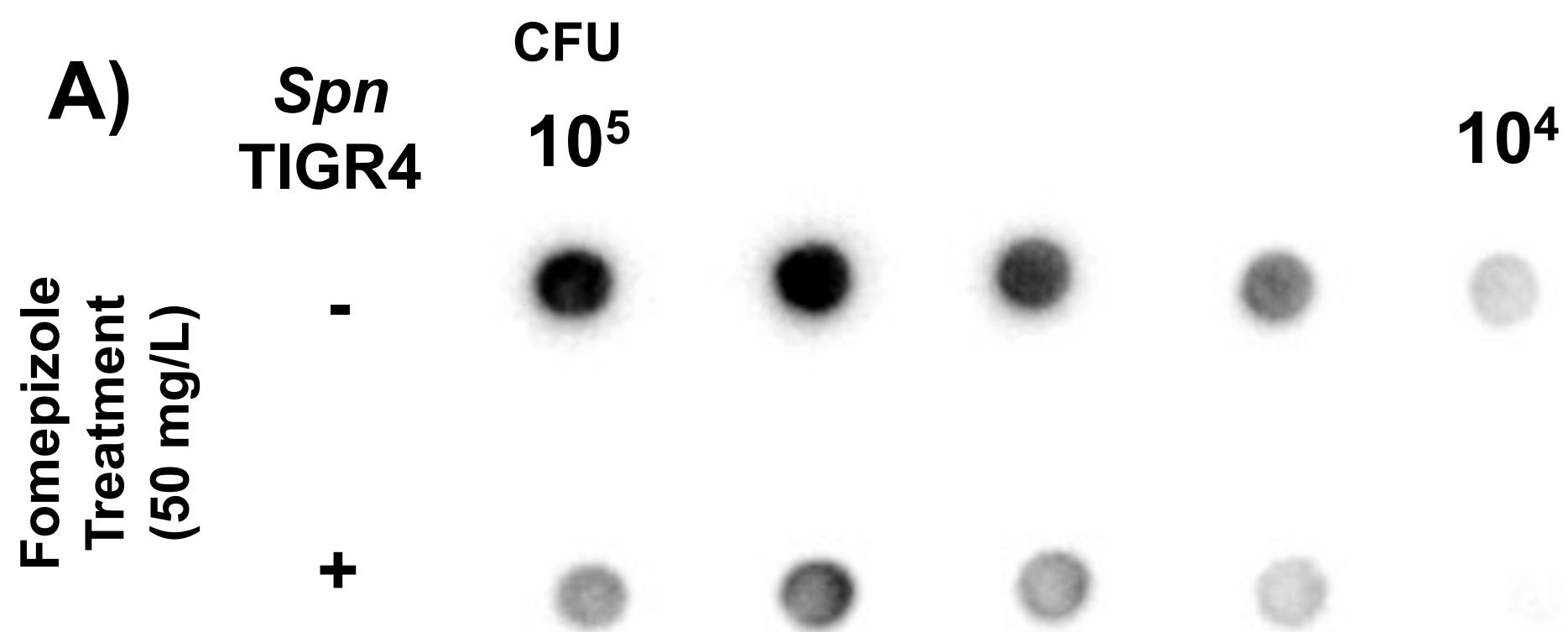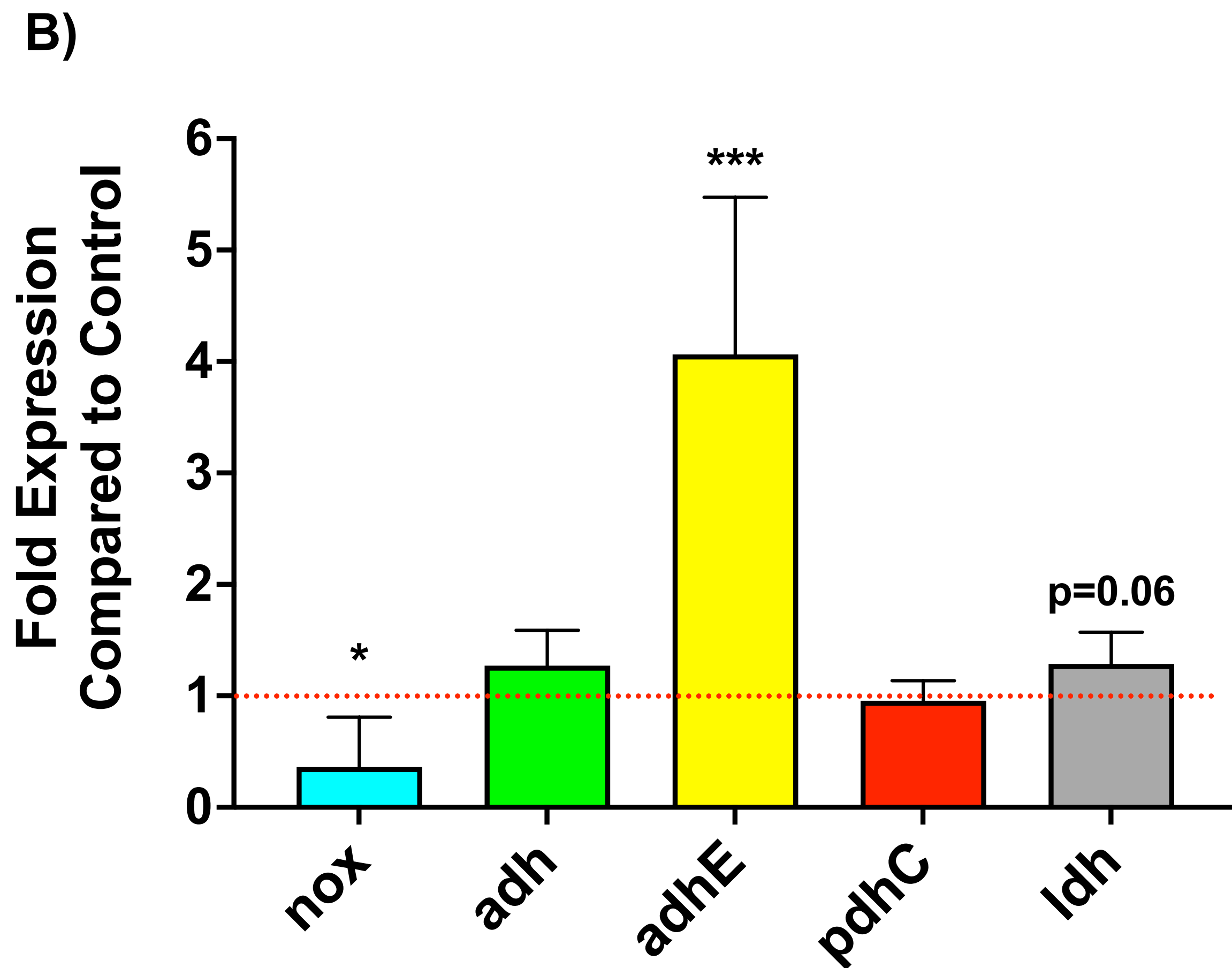

Supplement: S5 Fig — Fomepizole treatment decreased capsular polysaccharide production as determined by (A) immuno dot blot using type 4 specific anti-capsule antibody. (B) Altered gene expression of NAD+ regeneration-associated genes. In comparing and analyzing the gene expression level, the qRT-PCR result was normalized using rpoD as a reference. For both tests, fomepizole was applied to bacteria for 2 hours. The data underlying S5B Fig can be found in S1 Data. Mann–Whitney t tests between fomepizole and control groups were done. Asterisks indicate statistical significance: *, P ≤ 0.05, ***, P ≤ 0.001. (PDF) [file pbio.3002020.s005.pdf]

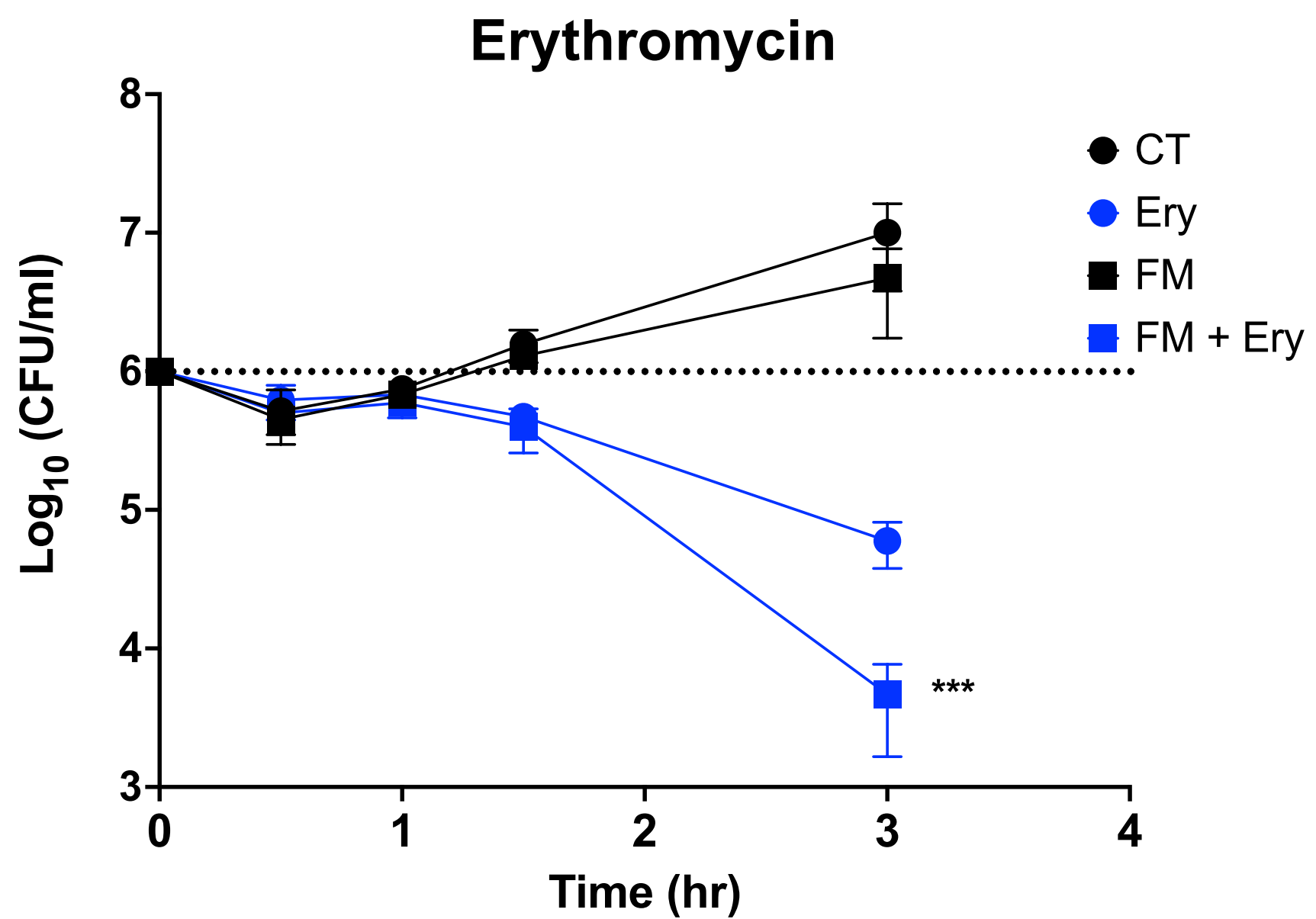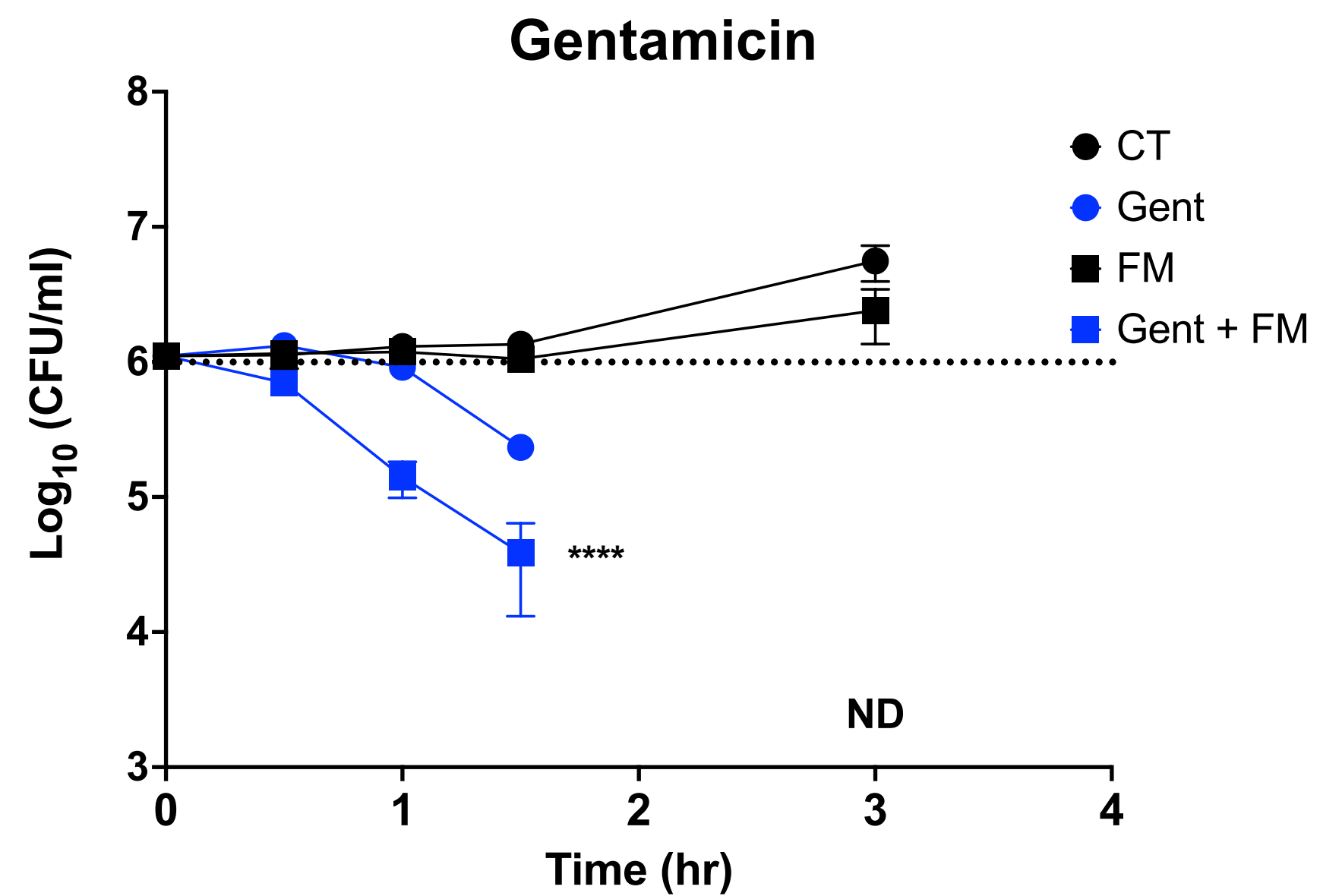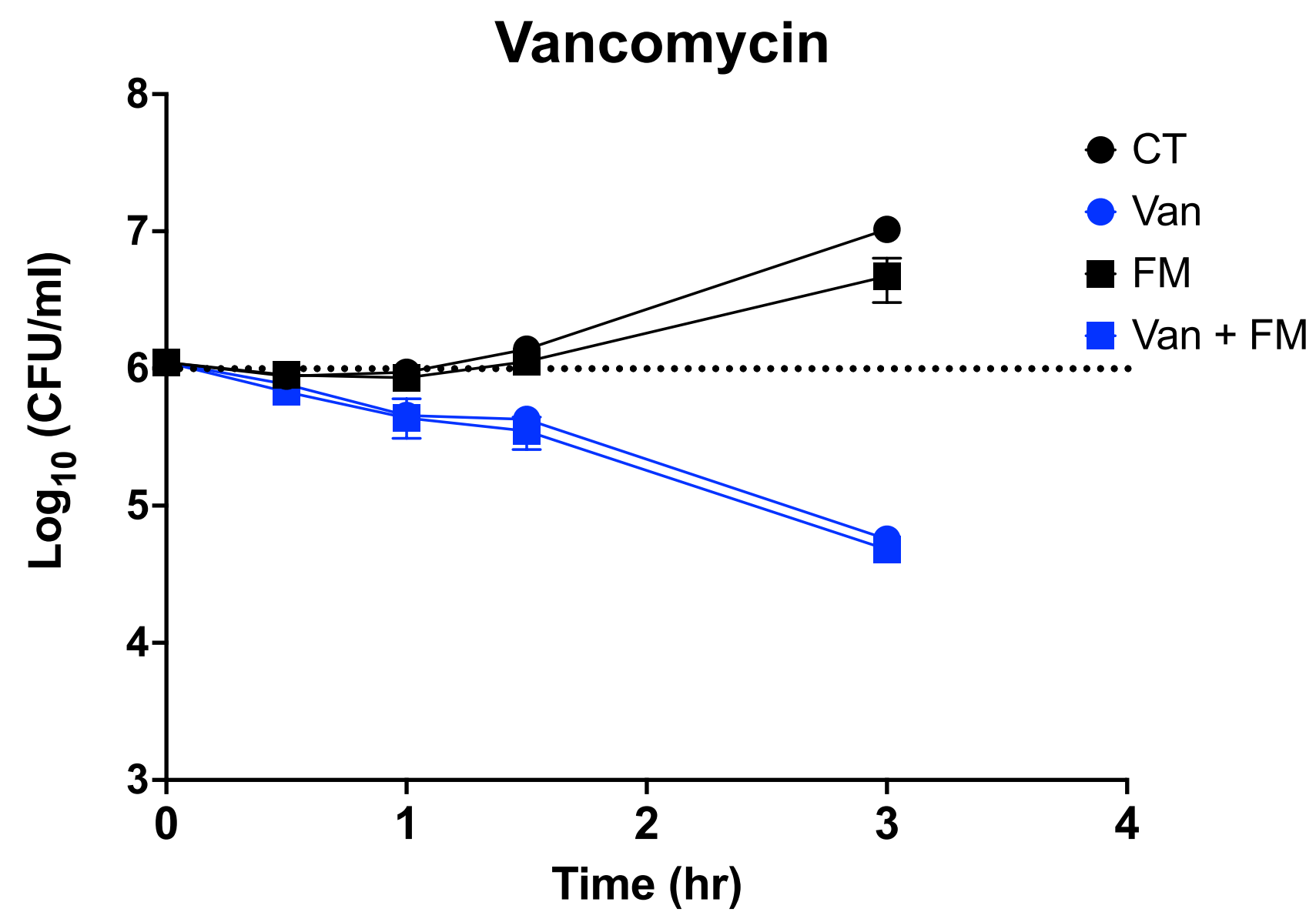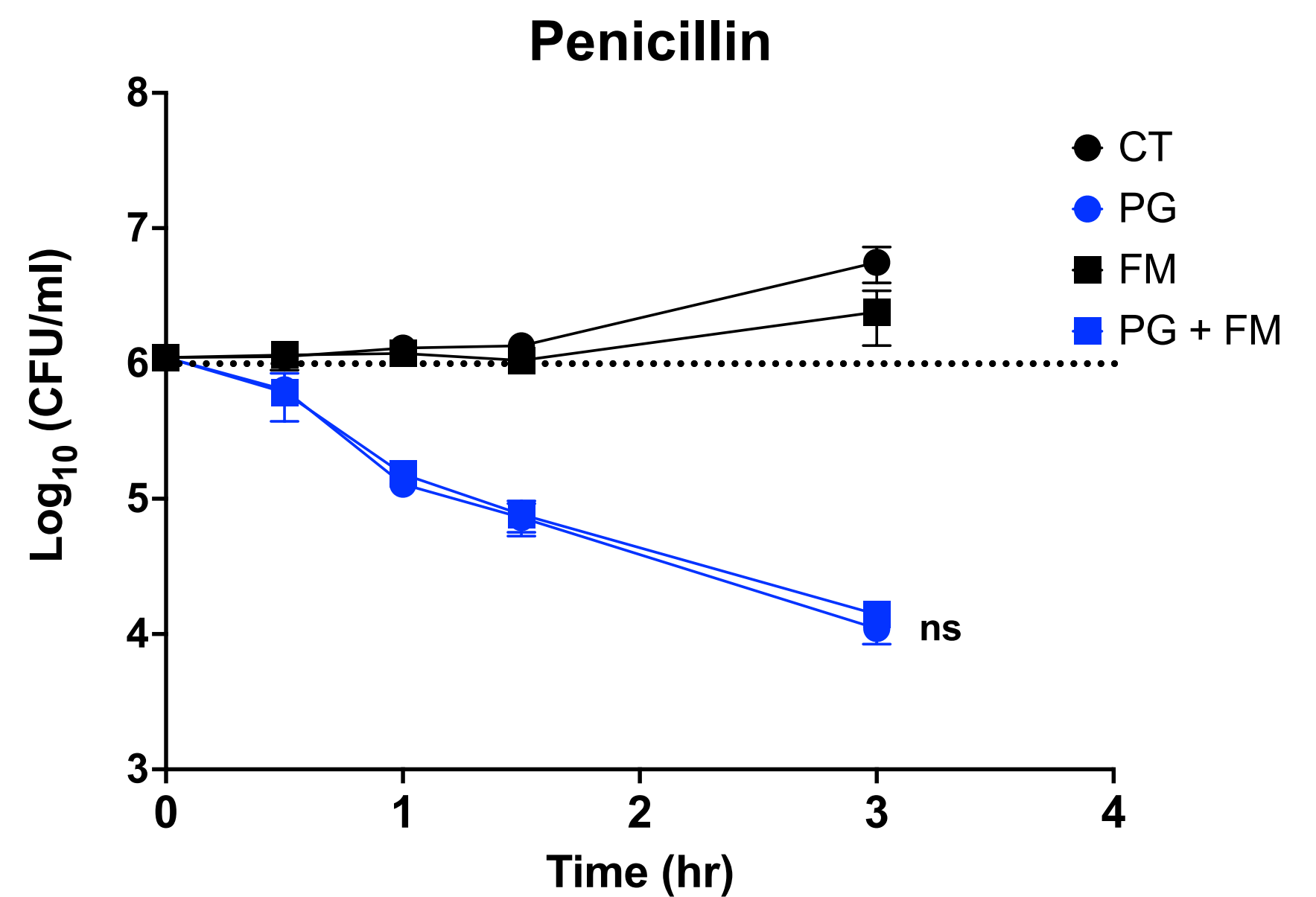

Supplement: S6 Fig — Antibiotic susceptibility was assessed using ribosome targeting antibiotics, erythromycin (5 μg/ml) and gentamicin (50 μg/ml), and cell wall synthesis inhibiting antibiotics, penicillin (1 μg/ml) and vancomycin (10 μg/ml) with and without fomepizole (FM). Cell viability was measured at the designated time points for 3 hours of incubation with antibiotics. The data underlying this figure can be found in S1 Data. Statistics were done with the Mann–Whitney t test between the antibiotics and a combination of fomepizole and antibiotic groups. Asterisks indicate statistical significance: ***, P ≤ 0.001, ****, P ≤ 0.0001. (PDF) [file pbio.3002020.s006.pdf]

*Spn* 35B  
162-5678

*Spn* TIGR4

1Kb

500bp

*ermB* *mef* *aphA* *ermB* *mef* *aphA*

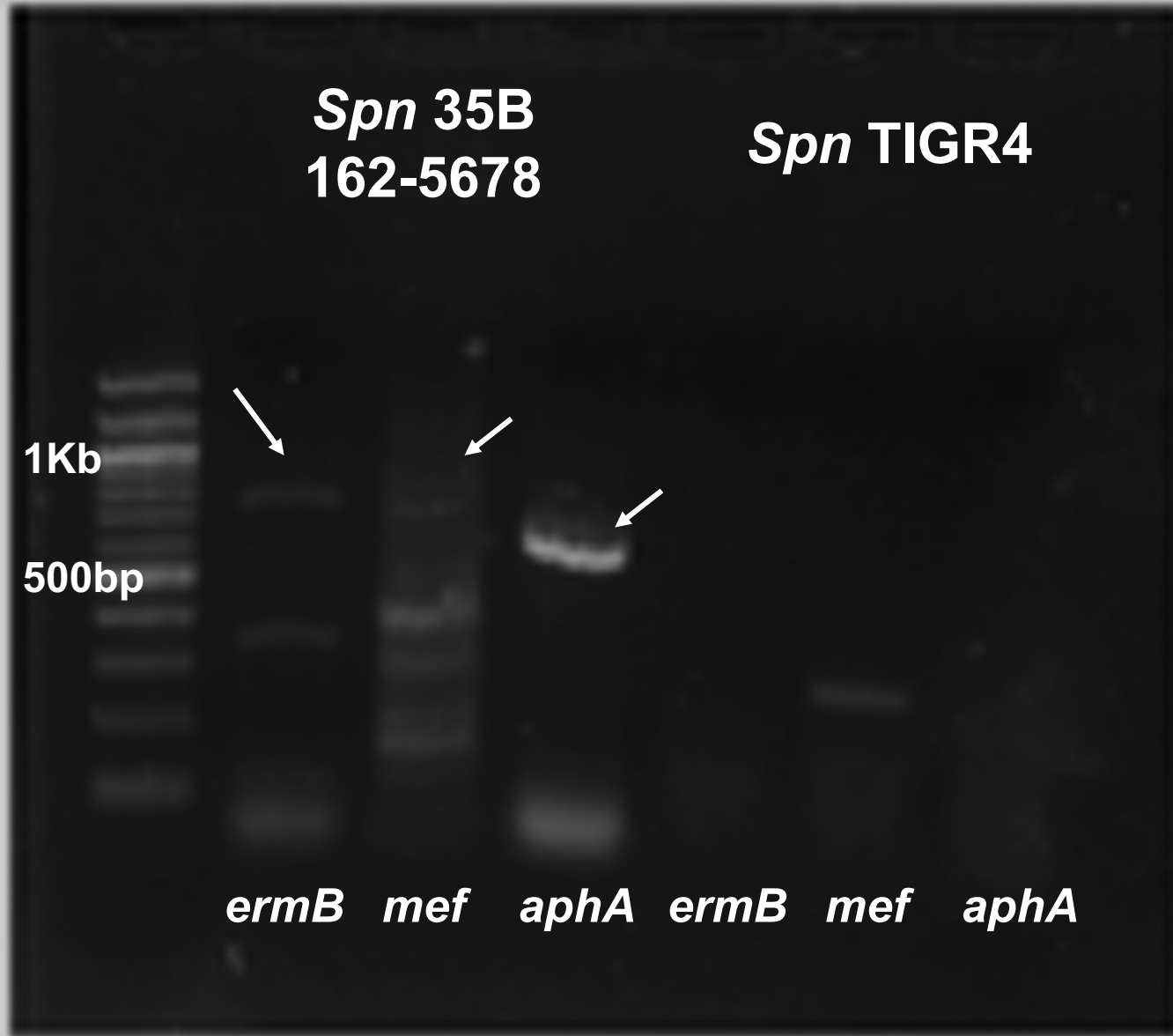

Supplement: S8 Fig — Primers were used for PCR detection of antibiotic resistance genes ermB, mef(A/E), and aphA3 in the 162–5678 chromosome. White arrows indicated the correct band of the PCR products for each gene. (PDF) [file pbio.3002020.s008.pdf]

A)

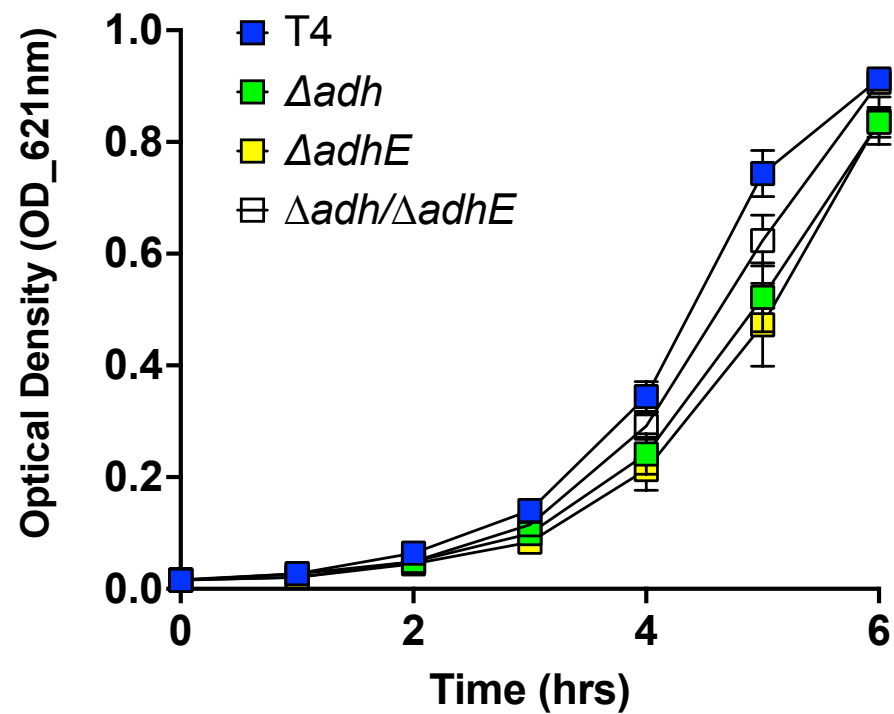

B)

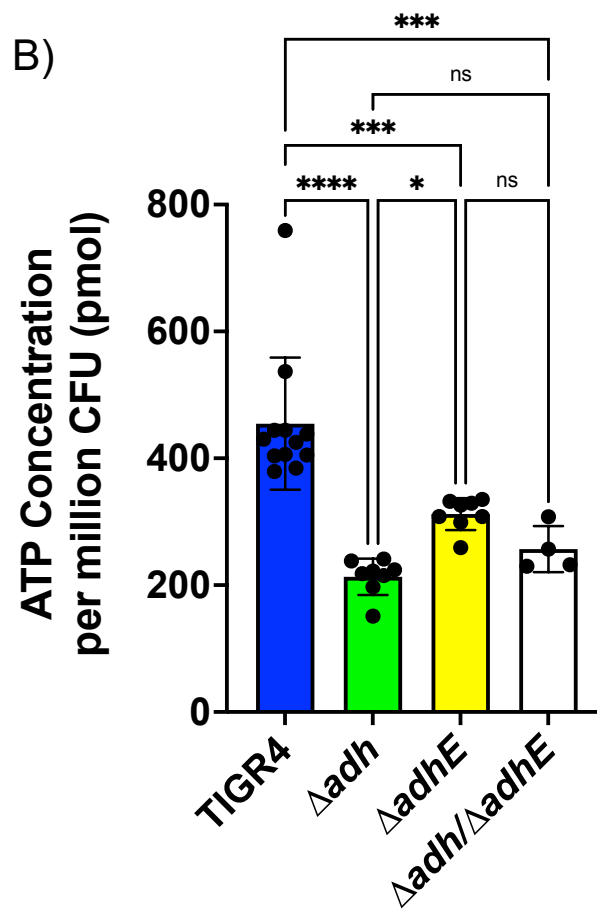

C)

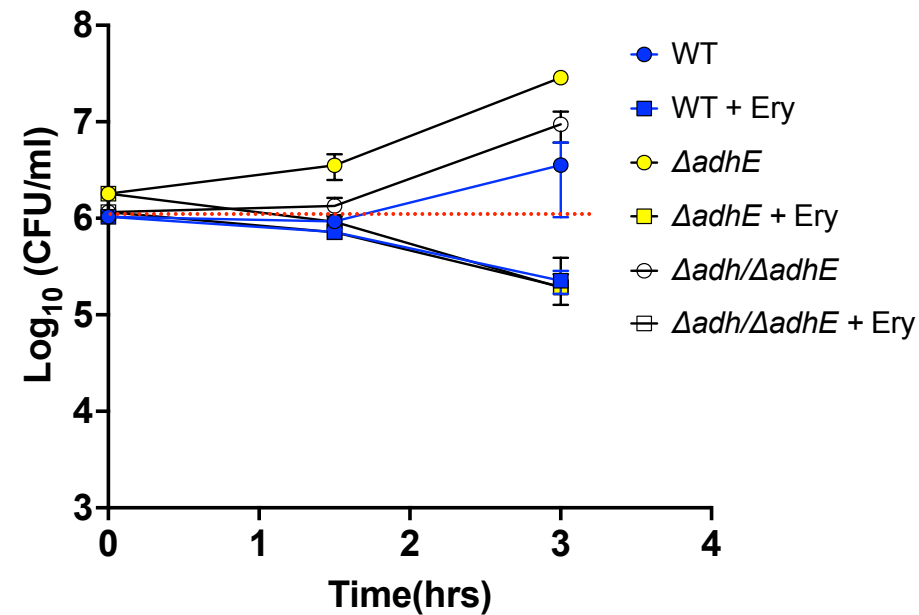

| <i>Spn</i> TIGR4    | WT           | <i>adh</i>   | <i>adhE</i>  | <i>adh/adhE</i> |
|---------------------|--------------|--------------|--------------|-----------------|
| Doubling Time (min) | 52.3<br>±3.2 | 59.1<br>±2.1 | 61.0<br>±4.6 | 53.9<br>±2.2    |

Supplement: S9 Fig — We generated the double knockout of the adh/adhE strain and evaluated its (A) growth rate, (B) intracellular ATP concentration, and (C) survival rate after erythromycin exposure. All assays were conducted as described in the Materials and methods. (n > 3) The data underlying this figure set can be found in S1 Data. Statistical analyses were done with the Mann–Whitney t test between TIGR4 WT. Asterisks indicate statistical significance: *, P ≤ 0.05, **, P ≤ 0.01; ***, P ≤ 0.001; ****, P ≤ 0.0001. (PDF) [file pbio.3002020.s009.pdf]
