# Supplementary material for: Targeting NAD+ regeneration enhances antibiotic susceptibility of Streptococcus pneumoniae during invasive disease
Source: PLoS Biol. 2023 Mar 16;21(3):e3002020. doi: 10.1371/journal.pbio.3002020 (PMC10019625; doi:10.1371/journal.pbio.3002020)
Supplement: S7 Fig — After 16–24 hours of incubation in THY media with designated amounts of fomepizole and antibiotics, viability of bacteria was assessed. At least 3 independent assays were performed (n ≥ 3). The data underlying this figure can be found in S1 Data. For Streptococcus, erythromycin was used as a model antibiotic, and gentamicin was used for E. faecium due to its high resistance to other antibiotics. (PDF) [file pbio.3002020.s007.pdf]

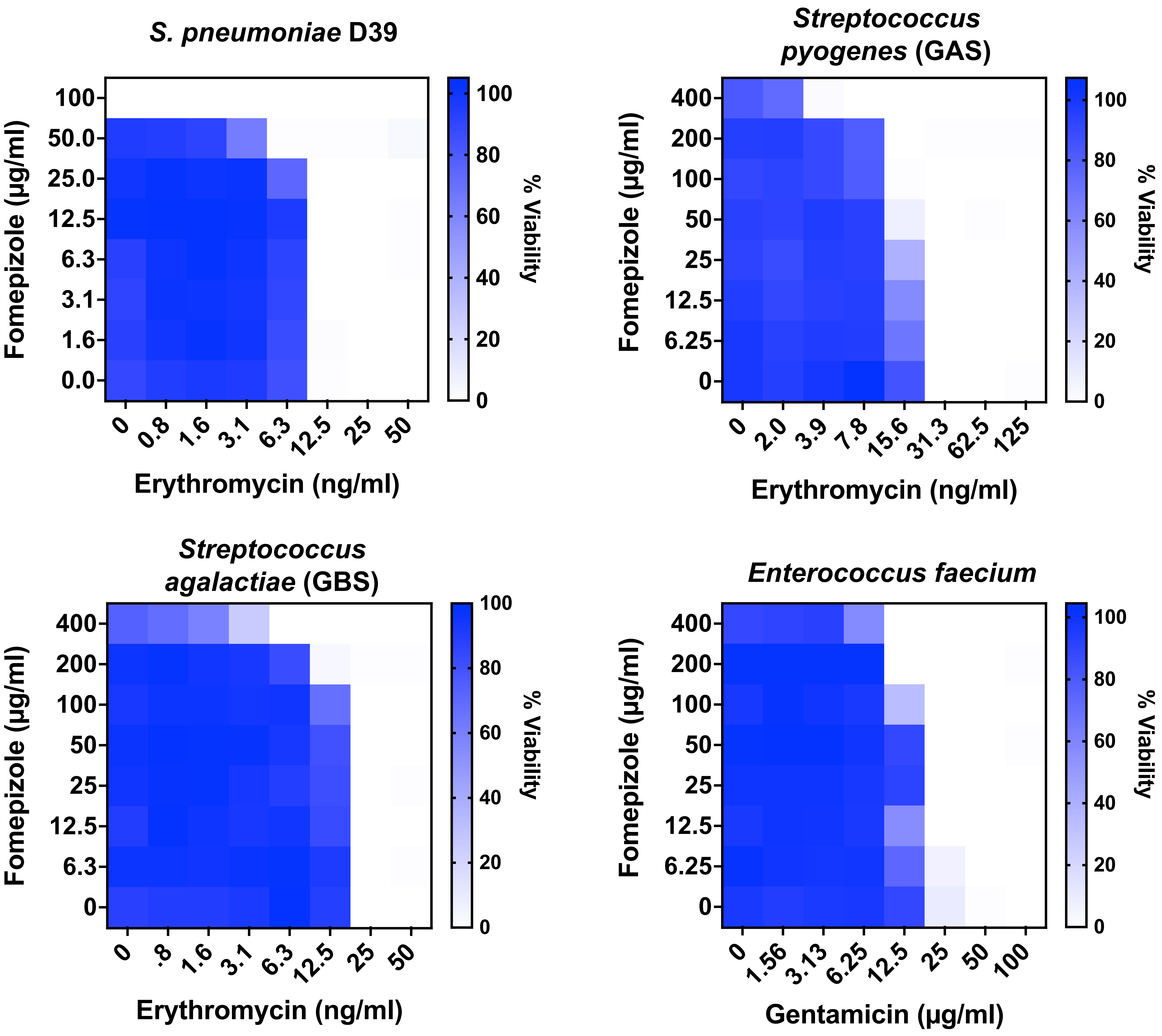

S. pneumoniae D39
Fomepizole (µg/ml)
100
50.0
25.0
12.5
6.3
3.1
1.6
0.0
0
0.8
1.6
3.1
6.3
12.5
25
50
Erythromycin (ng/ml)
% Viability
100
80
60
40
20
0
Streptococcus pyogenes (GAS)
Fomepizole (µg/ml)
400
200
100
50
25
12.5
6.25
0
0
2.0
3.9
7.8
15.6
31.3
62.5
125
Erythromycin (ng/ml)
% Viability
Streptococcus agalactiae (GBS)
Fomepizole (µg/ml)
400
200
100
50
25
12.5
6.3
0
0
.8
1.6
3.1
6.3
12.5
25
50
Erythromycin (ng/ml)
% Viability
Enterococcus faecium
Fomepizole (µg/ml)
400
200
100
50
25
12.5
6.25
0
0
1.56
3.13
6.25
12.5
25
50
100
Gentamicin (µg/ml)
% Viability
