# Supplementary material for: Targeting NAD+ regeneration enhances antibiotic susceptibility of Streptococcus pneumoniae during invasive disease
Source: PLoS Biol. 2023 Mar 16;21(3):e3002020. doi: 10.1371/journal.pbio.3002020 (PMC10019625; doi:10.1371/journal.pbio.3002020)
Supplement: S1 Table — (DOCX) [file pbio.3002020.s010.docx]

| **Bacterial Strain** | | **Description** | |
| --- | --- | --- | --- |
| *Spn* TIGR4 | | Wild type (Kan^S^/Sm^R^) | |
|  | ∆*nox* | *nox* (SP_1469) mutant in TIGR4, ∆*nox*::Kan, (Kan^R^/Sm^S^) |  |
|  | ∆*adh* | *adh* (SP_0285) mutant in TIGR4, ∆*adh*::Kan, (Kan^R^/Sm^S^) |  |
|  | ∆*adhE* | *adhE* (SP_2026) mutant in TIGR4, ∆*adhE*::Kan, (Kan^R^/Sm^S^) |  |
|  | ∆*pdhC* | *pdhC* (SP_1162-1164) mutant in TIGR4, ∆*pdhC*::Kan, (Kan^R^/Sm^S^) |  |
|  | ∆*ldh* | *ldh* (SP_1220) mutant in TIGR4, ∆*ldh*::Kan, (Kan^R^/Sm^S^) |  |
|  | ∆*nox::nox* | *nox* gene-complemented strain, ∆*nox::nox,* (Kan^S^/Sm^R^) |  |
|  | ∆*adh::adh* | *adh* gene-complemented strain, ∆*nox::nox,* (Kan^S^/Sm^R^) |  |
|  | ∆*adhE::adhE* | *adhE* gene-complemented strain, ∆*nox::nox,* (Kan^S^/Sm^R^) |  |
|  | ∆*pdhC::pdhC* | *pdhC* gene-complemented strain, ∆*nox::nox,* (Kan^S^/Sm^R^) |  |
|  | ∆*ldh::ldh* | *ldh* gene-complemented strain, ∆*ldh::ldh ,* (Kan^S^/Sm^R^) |  |
| *Spn* D39 | |  | |
| *Spn* 35B | | 162-5678 multi-drug resistant clinical isolate, gifted from Dr. Benjamin at UAB | |
| *Streptococcus pyogenes* | | Group A Streptococci | |
| *Streptococcus agalactiae* | | Group B Streptococci | |
| *Enterococcus faecium* | | Clinical isolate | |

**Table S1. Bacterial strains used in the study**

**^R^: Resistance**

**^S^: Susceptible**
