# Supplementary material for: Targeting NAD+ regeneration enhances antibiotic susceptibility of Streptococcus pneumoniae during invasive disease
Source: PLoS Biol. 2023 Mar 16;21(3):e3002020. doi: 10.1371/journal.pbio.3002020 (PMC10019625; doi:10.1371/journal.pbio.3002020)
Supplement: S2 Table — (DOCX) [file pbio.3002020.s011.docx]

**Table S2. Primers used in the study**

| **Bacteria/Purpose** | **Name** | **Primer sequences (5’-3’)** | |
| --- | --- | --- | --- |
| *S. pneumoniae* mutant construction | | | |
|  | Construction was done using Janus cassette^1,2^ from SpNTB112 (TIGRJS) | | |
| ∆*nox* mutation | Nox-1 | ttggatttcattttcattaataccaaccatggc | |
|  | Nox-2 | tcaaacggatcgatccttaaatcacaatggctgcccttacg | |
|  | Nox-3 | gtaagggcagccattgtgatttaaggatcgatccgtttgatttttaatggataatgt | |
|  | Nox-4 | tcgttgtagtcggtgctaacttatgcttttggacgtttagtaccgtatttaga | |
|  | Nox-5 | ctaaacgtccaaaagcataagttagcaccgactacaacgattttactc | |
|  | Nox-6 | atgtgatggtaatcttgtccatcgacg | |
| ∆*adh* mutation | Adh-1 | aagggcatgtgtttctccaaaacg | |
|  | Adh-2 | tcaaacggatcgatccttaagaaaaaggccaaatccaaggacgt | |
|  | Adh-3 | ccttggatttggcctttttcttaaggatcgatccgtttgatttttaatggataatgt | |
|  | Adh-4 | atccagaaagcactggtgttttatgcttttggacgtttagtaccgtatttagaac | |
|  | Adh-5 | ctaaacgtccaaaagcataaaacaccagtgctttctggatttacaac | |
|  | Adh-6 | agatttttaccgtgaactgggcttagac | |
| ∆*adh*E mutation | AdhE-1 | tatcaaaataaataggaacactgtcattcccatctg | |
|  | AdhE-2 | tcaaacggatcgatccttaatacaaagaaagaccaggacgcc | |
|  | AdhE-3 | cgtcctggtctttctttgtattaaggatcgatccgtttgatttttaatggataatg | |
|  | AdhE-4 | aaaaaactgtgacaccagagttatgcttttggacgtttagtaccgtatttagaacg | |
|  | AdhE-5 | ctaaacgtccaaaagcataactctggtgtcacagtttttttatcagcc | |
|  | AdhE-6 | ggggactcagggaatttttcaagagt | |
| ∆*pdhC* mutation | pdhC-1 | tggccaacttctttttgatttatccaactacc | |
|  | pdhC-2 | agcattatccggtacctctcctaggaatggccatccattcacc | |
|  | pdhC-3 | gaatggatggccattcctaggagaggtaccggataatgctgaaaact | |
|  | pdhC-4 | tagataaaaatcttttgctagagactcgagcctttccttatgct | |
|  | pdhC-5 | taaggaaaggctcgagtctctagcaaaagatttttatctaaagttgacatcttcttgc | |
|  | pdhC-6 | ctcaatagcggatttaactatctcttgatttacgg | |
| ∆*ldh* mutation | ldh-1 | cttggttttattgaccatgtaaggaaattctgt | |
|  | ldh-2 | agcattatccggtacctctcaacttttttgtgttgtttagttgaagtcattgt | |
|  | ldh-3 | ctaaacaacacaaaaaagttgagaggtaccggataatgctgaaaact | |
|  | ldh-4 | tcttggaattctgggtttttgagactcgagcctttccttatgct | |
|  | ldh-5 | taaggaaaggctcgagtctcaaaaacccagaattccaagaagcttct | |
|  | ldh-6 | tttacgagggtttgcaagaacaattcc | |
| qRT-PCR primers | Adh rt-F | aaagttgaaccaggccaatg | |
|  | Adh rt-R | cgcctacttcttttgcaagg | |
|  | adhE rt-F | tgctcctgaaaactgtgtgc | |
|  | adhE rt-R | acctaccccaagagctggtt | |
|  | ldh rt-F | ttggtgcacatggtatcgtt | |
|  | ldh rt-R | tctgggtttttccatgcttc | |
|  | pdhC rt-F | tcgtgtccacaaagaagacg | |
|  | pdhC rt-R | gatttcctgccaagcaatgt | |
|  | nox rt-F | ctgttggtgactgtgcgact | |
|  | nox rt-R | tgtaggcaccaacgatacca | |
|  | rpoD rt-F | ggtcgtggtatgcagttcct | |
|  | rpoD rt-R | cacgagtgatagcctgacga | |
|  | erm(B)-F | tggtattccaaatgcgtaatg | Reference^3^ |
|  | erm(B)-R | ctgtggtatggcgggtaagt |  |
|  | mef(A/E)-F | caatatgggcagggcaag |  |
|  | mef(A/E)-R | aagctgttccaatgctacgg |  |
|  | aphA3-F | taa aag ata cgg aag gaa tgt ctc |  |
|  | aphA3-R | tcg acc gga cgc aga agg caa tgt |  |

**Reference**

1 Brissac, T. *et al.* Capsule Promotes Intracellular Survival and Vascular Endothelial Cell Translocation during Invasive Pneumococcal Disease. *mBio* **12**, e0251621, doi:10.1128/mBio.02516-21 (2021).

2 Hollands, A. *et al.* Genetic switch to hypervirulence reduces colonization phenotypes of the globally disseminated group A streptococcus M1T1 clone. *J Infect Dis* **202**, 11-19, doi:10.1086/653124 (2010).

3 Talebi, M. *et al.* Determination of Characteristics of Erythromycin Resistant Streptococcus pneumoniae with Preferred PCV Usage in Iran. *PLoS One* **11**, e0167803, doi:10.1371/journal.pone.0167803 (2016).
