# Supplementary material for: Targeting NAD+ regeneration enhances antibiotic susceptibility of Streptococcus pneumoniae during invasive disease
Source: PLoS Biol. 2023 Mar 16;21(3):e3002020. doi: 10.1371/journal.pbio.3002020 (PMC10019625; doi:10.1371/journal.pbio.3002020)

Figure 2A raw images

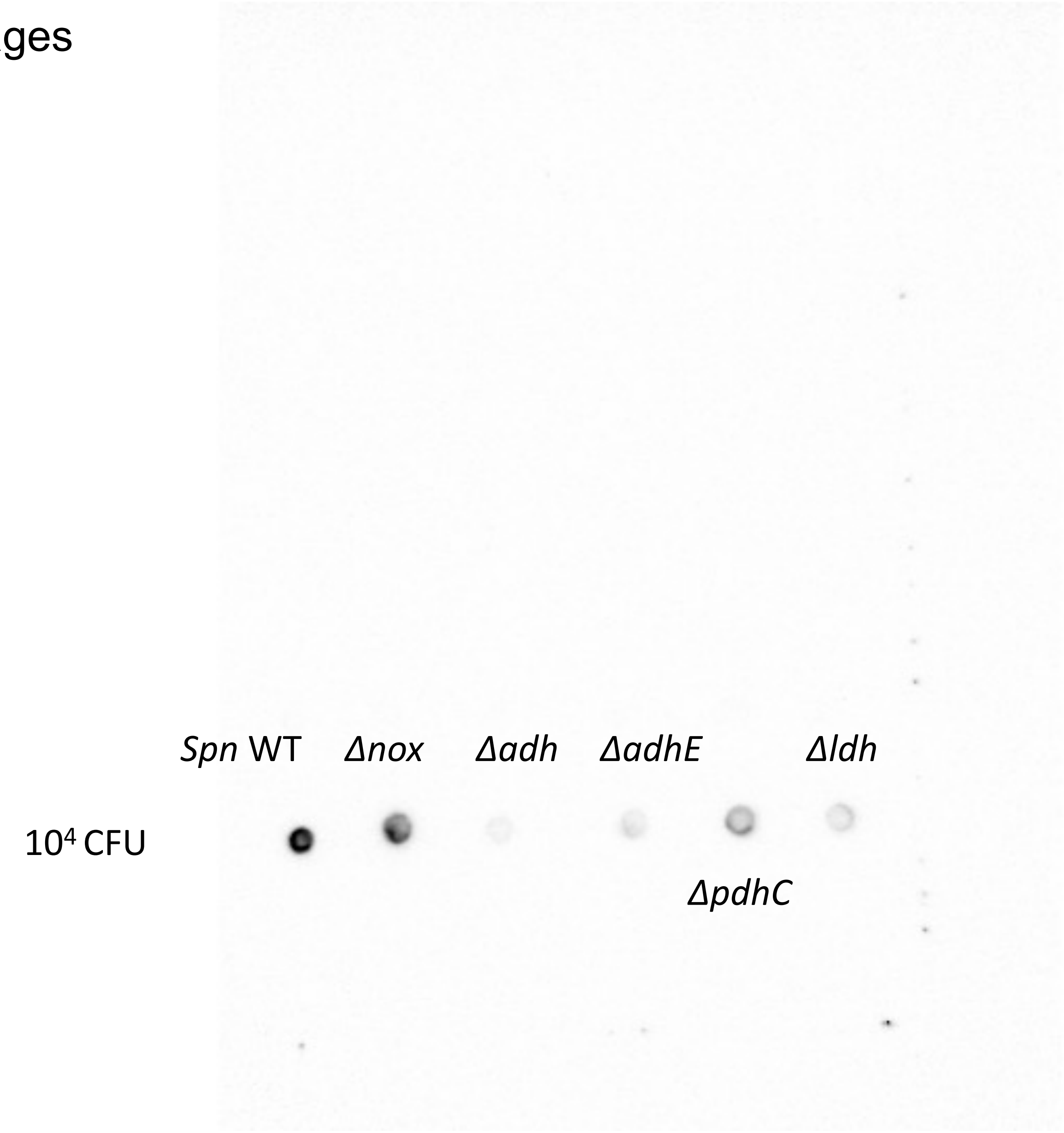

Figure 2B Pneumolysin and PspA original blot

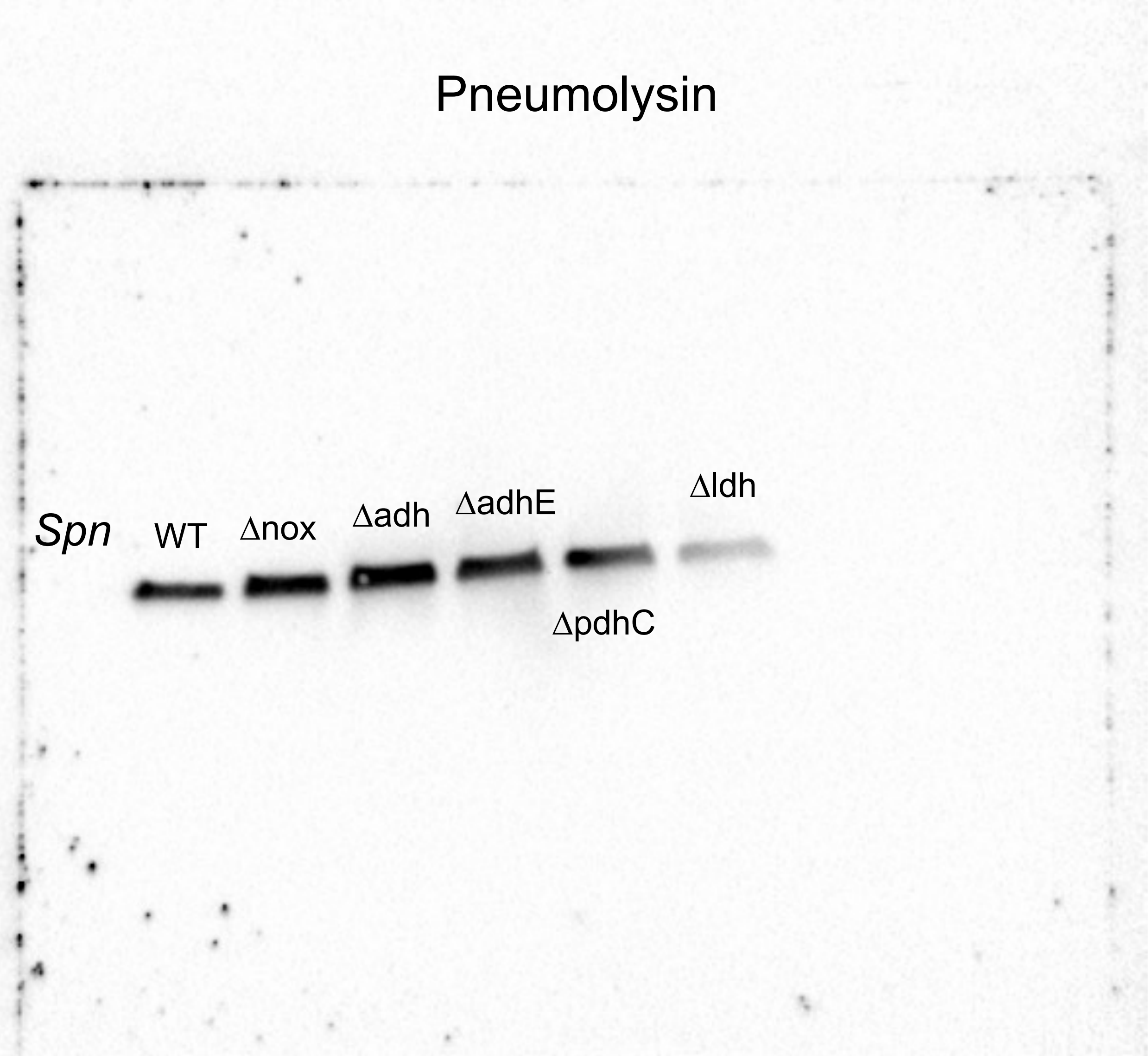

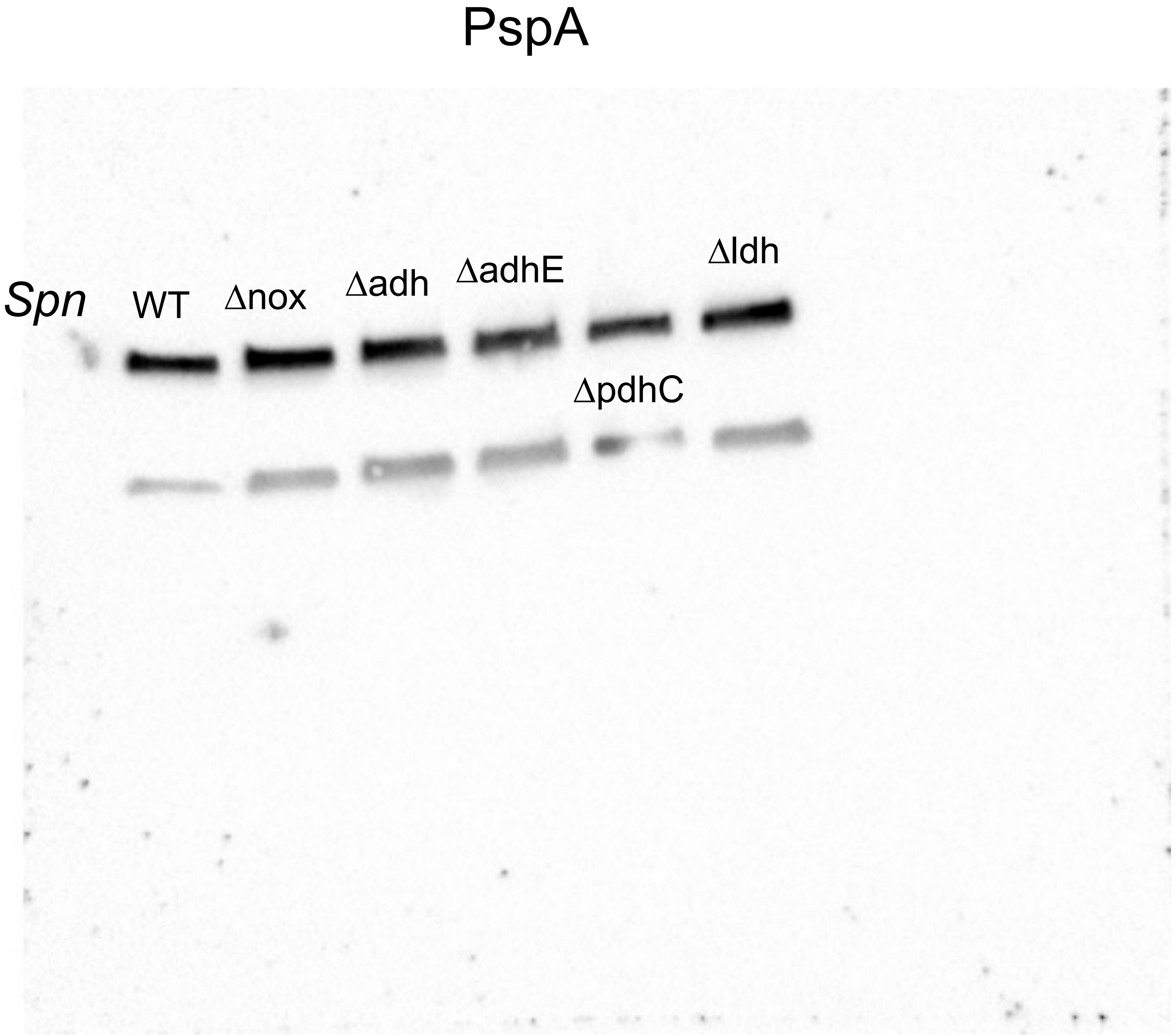

Figure 2B Pneumolysin original blot

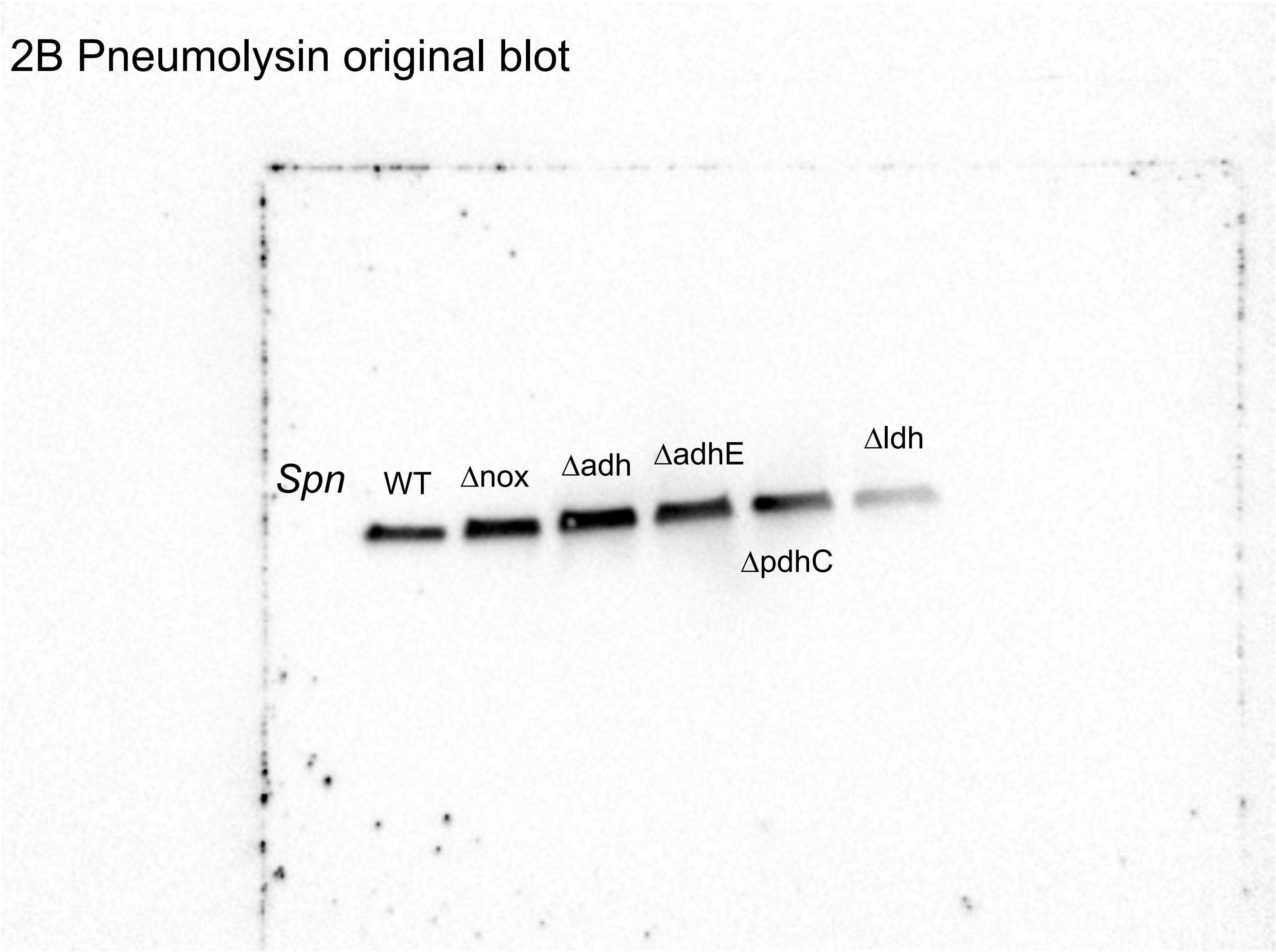

Figure S5 Original blot image

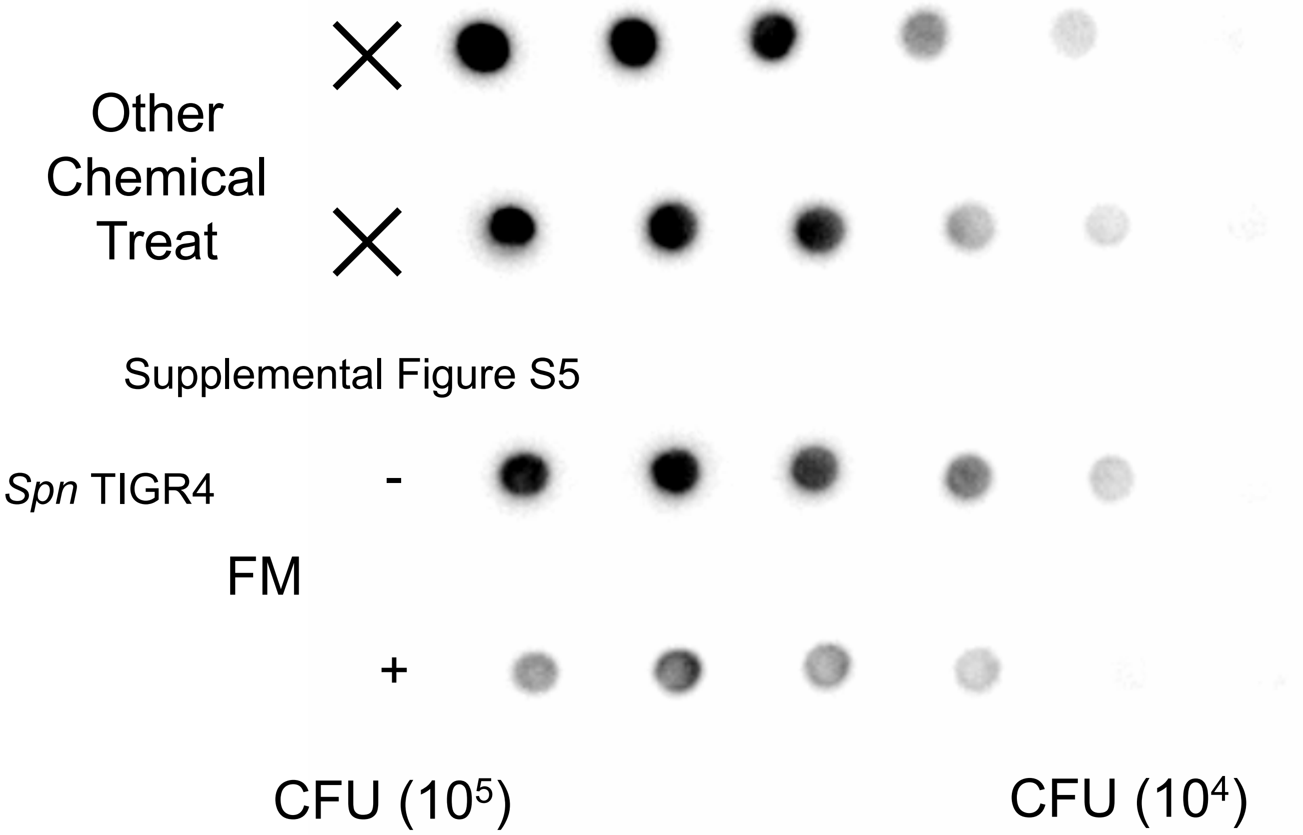

Figure S8 Original raw image

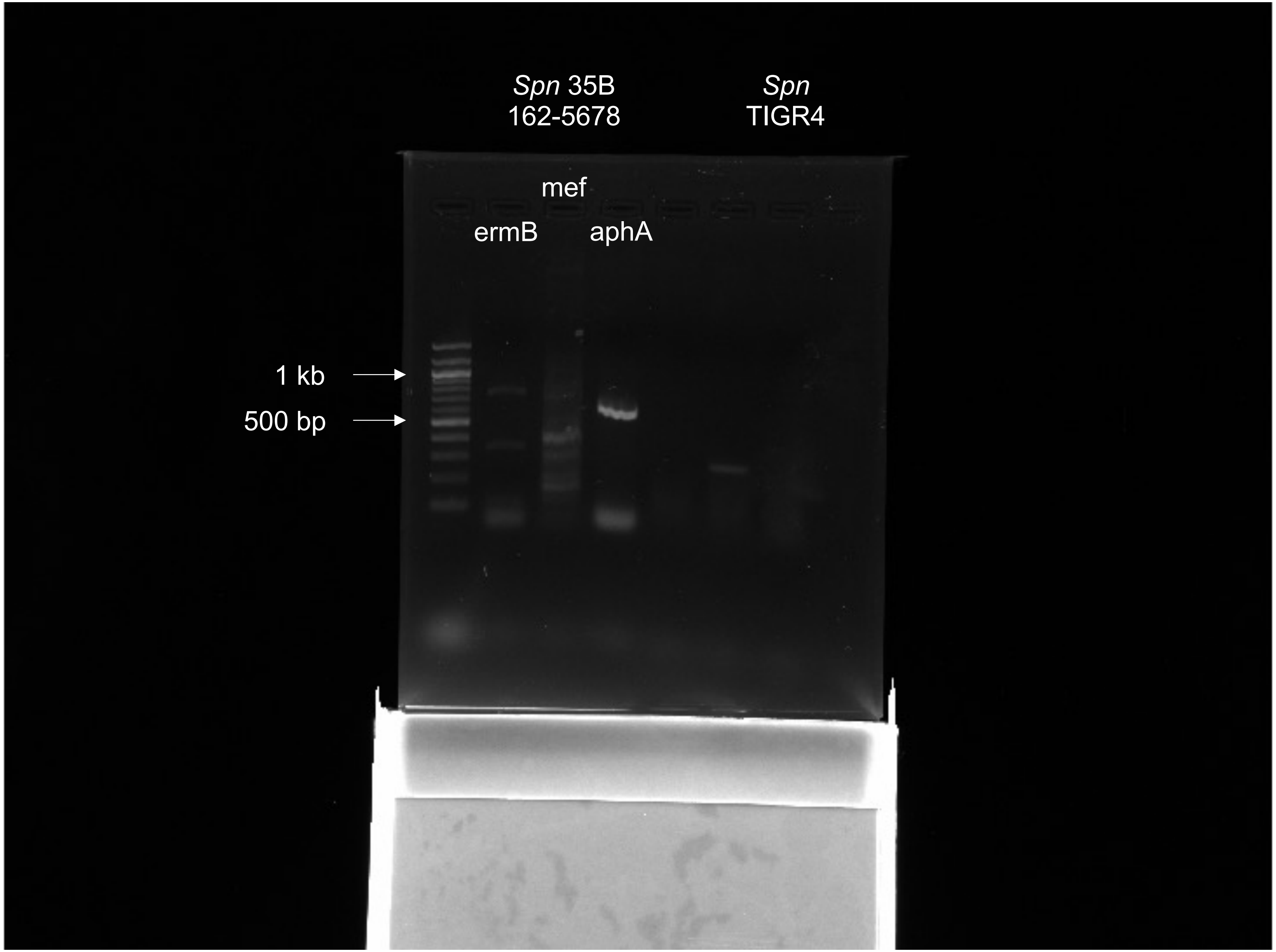

Supplement: S1 Raw Images — This file has the raw image of Figs 2A and 2B, S5 and S8. (PDF) [file pbio.3002020.s013.pdf]
