# Supplementary material for: Targeting NAD+ regeneration enhances antibiotic susceptibility of Streptococcus pneumoniae during invasive disease
Source: PLoS Biol. 2023 Mar 16;21(3):e3002020. doi: 10.1371/journal.pbio.3002020 (PMC10019625; doi:10.1371/journal.pbio.3002020)
Supplement: Attachment — Submitted filename: Responses to the Reviewers comments 1221_Final.docx [file pbio.3002020.s014.docx]

**Reviewer’s comment and answers**

**Editor:**

As you will see below, the reviewers find your work interesting but they find issues that would need to be solved before publication. We think it is important that you address the technical points from reviewer #1, in particular the need for complementation assays to validate gene deletion phenotypes. We think it is essential that you include control experiments as requested, and experimental revision to address points 4-6 of reviewer #1 and to points 1 and 2 of reviewer #2.

**Answer:** We thank the editor or the opportunity to revise and resubmit the manuscript. As is detailed below, we have responded to the reviewer’s major concerns. This includes the creation of revertant strains for our mutants and their testing. We have also added the additional animal experiments at other anatomical sites as requested by Reviewer 2. These changes, along with those addressing minor points, have improved the manuscript.

**Reviewer #1:**

This is an interesting and potentially important paper in view of the current increase in macrolide resistance in *Streptococcus pneumoniae*. The finding that blocking the redox-balance enhances pneumococcal susceptibility to antibiotics that inhibit ribosome functions, suggest the use of redox modulators, such a inhibitors of alcohol-dehydrogenases as antibiotic potentiators. However, the paper would be stronger and more convincing by addition of some control experiments.

**Answer:** We thank the reviewer for their constructive critiques. We have added the

requested control experiments and believe that our conclusion are further supported and

the manuscript stronger.

Comments:

1. The main message is that inhibition of alcohol dehydogenase with 4-methylpyrazole sensitizes macrolide resistant pneumococci to erythromycin in an animal treatment model. It would be good to add some sentences about the current situation of beta-lactam and macrolide resistance in Streptococcus pneumoniae as these are the two main classes of antibiotics used to treat pneumococcal infections.

**Answer:** Thank you for the comment. We have added sentences to the introduction where we describe the general extent of the antibiotic-resistance problem and the current situation with Streptococcus pneumoniae from Ln 39~44.

2. A panel of mutants were generated in a set of pneumococcal genes affecting redox balance. Since they were constructed by inserting a kanamycin resistance casette, polar effects on down stream genes cannot be excluded. To be sure that the phenotype is caused by inactivation of the target gene, complementation needs to be done. Also, the kanamycin resistance casette may cause a negative effect in competition experiments with the wild-type (lacking the casette).

Answer: This is a valid concern. Unfortunately, *S. pneumoniae* does not tolerate plasmids, making complementation of mutants difficult. This issue is aggravated by the fact that we have access to only one version of the mutagenic Janus cassette – which confers kanamycin resistance. Thus, we currently do not have the capacity to create a complemented mutant *per se*, but could create a revertant, where the mutated loci is restored to wildtype. Briefly, to construct a revertant strain, the amplicon of the original gene is transferred back to the site where the Janus cassette is inserted according to the below references. The following studies used revertants for their complementation assays.

- Epigenetic Switch Driven by DNA Inversions Dictates Phase Variation in *Streptococcus pneumoniae*. Plos Pathogen. (2016).

- Role of *Streptococcus pneumoniae* OM001 operon in capsular polysaccharide production, virulence, and survival in human saliva. Plos One. (2018)

Revertants were created using all our mutants, and when tested, these strains behaved identical to wildtype, indicating that the phenotype was due to disruption of the gene/locus of interest and not due to offsite alteration of the genome. This new data is included in the manuscript. Importantly, our conclusions are only in part dependent on genetic deletion of loci of interest. We support our results with pharmacological blocking of alcohol dehydrogenase activity in wildtype – which resulted in near identical results as when alcohol dehydrogenases were deleted.

3. Is the erythromycin dose given to the animals similar to the dose given for humans?

Answer: We used 25 mg/kg concentration for the test, which was in the range to treat patients, including children. We added a sentence in our result for the explanation at Ln 185-186 with a reference.

4. Macrolide resistance can be caused by mechanisms such as modification of the target and/or efflux. It is not evident that the alcohol-dehydrogenase inhibitor potentiates erythromycin for both types of resistancies.. It is easy to determine which resistance genes are present in the clinical 35B isolate used in this study.

Answer: Thank you for the great idea. We conducted a PCR test for Spn 35B serotype with ErmB, efflux pump (mef), and macrolide resistance (aphA3) primers to detect the presence of antibiotic-resistant genes as described in Talebi et al. (2016). We included this result as Supplemental Figure 8 and have added a sentence in the main text with this reference.    

5. The mechanisms explaining the increased sensitivity of the redox mutants to ribosome acting antibiotics has not been elucidated in this paper. Can something more on this topic be added to the paper?

Answer: Thank you for your comment. As the reviewer recommended, we examined efflux pump activity using our panel of mutants. We found that all the mutants had reduced efflux capacity compared to the parental strain. We added those results in Figure 4 and have added a paragraph discussing this to the main text. We suspect that there are likely other reasons. However, more detailed studies on the underlying mechanisms fall outside the scope of this manuscript, whose purpose was to determine if there was an effect.

6. Pneumococci have two genes encoding alcohol dehydrogenases. What is the phenotype when both these genes have been inactivated?

Answer: We constructed a double knockout of *adh*/*adhE* and evaluated their phenotypes using growth rate, ATP concentration, and antibiotic resistance. Interestingly, we found that double knockout strains behaved similarly to adhE mutants, which was not expected. Since all metabolic pathways mutually interact, we could not specify why, but we postulate in the discussion that the major form of compensation is through other metabolic pathways. The new data is part of Supplemental Figure S9.

7. Change ng/ml to microgram/ml for all. In Table 1 there is some mixup since I do not think D39 is resistant to erythromycin.

Answer: We apologize for this mistake. We revised all the unit with microgram/ml

Reviewer #2: [1] The authors provide a very brief explanation as to why capsule size is reduced in all of the mutants, but this could do with some more detail and expansion. It is taken as read that reduced capsule size means less virulent, but it would be interesting to test each of the mutants in both IV blood infection and upper respiratory tract colonization models. Reduction in capsule size may not necessarily mean reduced survival and or colonisation rate.

Answer: Thank you for the comment. As reviewer recommended, we evaluated the competitive index during nasal colonization. Using this model, we observed that the majority of our mutants colonized at an enhanced rate as wildtype. Given that they express lower levels of capsule (not none), this is not a surprising result as low-encapsulated pneumococci have enhanced fitness for colonization. The reason for this are explained in the text. Importantly, same phenotype confers susceptibility to opsonophagocytosis in pneumonia model, now also explained.

After much consideration, we decided against performing i.v. infection. The reason for this is that recent work done by our collaborator, Dr. Marco Oggioni, has shown that bacteria introduced to the blood are cleared by splenic macrophages [eBIoMedicine, 2021 Oct;72:103601]. In this model, sepsis occurred after challenge, but as result of bacterial re-emergence from splenic macrophages that were instead killed by the pneumococci that they had taken up. This model is therefore multi-step and complicated, and we were unsure what the metabolic fitness costs of our mutations would be on survival of pneumococci within macrophages and how data that emerges from such an experiment could be meaningfully interpreted.

[2] The pneumolysin - lactate dehydrogenase connection is also interesting. Could the authors expand on this? What are the clinical implications for example?

Answer: As shown in our colonization model, we found mutation of the lactate dehydrogenase showed dominance in the colonization model which is similarly shown in pneumolysin knockout strains. Compared to other strains, *ldh* mutants showed clear dominance for around 2 weeks. See our explanation immediately above.

[3] Figure 5, lung CFU data is interesting. There are clearly two distinct CFU groups in control group of mice, one clustered around Log 4 another at Log 7. Do the authors have an explanation for this? I have not seen this type of spread in CFU before. Is it possible that that the lower CFU clustering represents the slower growing lung CFU from the initial intratracheal admin and the higher CFU cluster, those bacteria which came back in from blood infection? It would have been good to see blood CFU data over time to see when the bacteria translocated across to blood and how CFUs behaved there over time.

Answer: Thank you for the comment. It is our experience that there are conditions where experimental challenge of mice results in a phenotype that appears to be bimodal or highly spread out. We propose that this is because the bacterium in some, but not all, instances fails to reach the critical mass necessary to sustain itself, and therefore is kept in check by the host. The blood CFU behavior is great idea, but we felt the scope of the work needed to rigorously address the question of how metabolically challenged pneumococci manage the translocation process was beyond the scope of this already large paper.
