# Supplementary material for: Targeting NAD+ regeneration enhances antibiotic susceptibility of Streptococcus pneumoniae during invasive disease
Source: PLoS Biol. 2023 Mar 16;21(3):e3002020. doi: 10.1371/journal.pbio.3002020 (PMC10019625; doi:10.1371/journal.pbio.3002020)
Supplement: Attachment — Submitted filename: Reviewer comment and answer.docx [file pbio.3002020.s015.docx]

Reviewer #1: The revised version of the manuscript has addressed all previous comments. I still think however that Table I needs revison.

Answer: We revised all units in the table to microgram/ml as you recommended following initial review.
